# The relationship between puberty and social emotion processing

**DOI:** 10.1111/j.1467-7687.2012.01174.x

**Published:** 2012-11

**Authors:** Anne-Lise Goddings, Stephanie Burnett Heyes, Geoffrey Bird, Russell M Viner, Sarah-Jayne Blakemore

**Affiliations:** 1 UCL Institute of Child HealthLondon UK; 2 UCL Institute of Cognitive NeuroscienceLondon, UK; 3 UCL Institute of NeurologyLondon, UK; 4 Department of Experimental PsychologyUniversity of Oxford, UK; 5 Department of Psychological SciencesBirkbeck College London, UK

## Abstract

The social brain undergoes developmental change during adolescence, and pubertal hormones are hypothesized to contribute to this development. We used fMRI to explore how pubertal indicators (salivary concentrations of testosterone, oestradiol and DHEA; pubertal stage; menarcheal status) relate to brain activity during a social emotion task. Forty-two females aged 11.1 to 13.7 years underwent fMRI scanning while reading scenarios pertaining either to social emotions, which require the representation of another person’s mental states, or to basic emotions, which do not. Pubertal stage and menarcheal status were used to assign girls to early or late puberty groups. Across the entire sample, the contrast between social versus basic emotion resulted in activity within the social brain network, including dorsomedial prefrontal cortex (DMPFC), the posterior superior temporal sulcus, and the anterior temporal cortex (ATC) in both hemispheres. Increased hormone levels (independent of age) were associated with higher left ATC activity during social emotion processing. More advanced age (independent of hormone levels) was associated with lower DMPFC activity during social emotion processing. Our results suggest functionally dissociable effects of pubertal hormones and age on the adolescent social brain.

## Introduction

Adolescence is a key stage in human development, incorporating physical, social, and psychological changes, and culminating in the attainment of a stable adult role (Lerner & Steinberg, 2004). Increasingly, adolescence is understood not merely as a transition between childhood and adulthood, but as a critical developmental period. During adolescence, new behaviours are laid down, educational, socioeconomic and relationship trajectories are canalized, and a new epidemiology of disease burden emerges (Patton & Viner, 2007). Many of these changes have been linked with puberty, the biological process that culminates in reproductive competence and a defining event of adolescence (Sisk & Foster, 2004).

Longitudinal and cross-sectional magnetic resonance imaging (MRI) studies have shown that the brain undergoes substantial structural remodelling during adolescence (Lenroot & Giedd, 2006; Shaw, Kabani, Lerch, Eckstrand, Lenroot, Gogtay, Greenstein, Clasen, Evans, Rapoport, Giedd & Wise, 2008). Converging evidence suggests that some of these changes may be related to increasing pubertal (i.e. adrenal and gonadal) hormones (Peper, Hulshoff, Pol, Crone & van Honk, 2011; Perrin, Hervé, Leonard, Perron, Pike, Pitiot, Richer, Veillette, Pausova & Paus, 2008; Perrin, Leonard, Perron, Pike, Pitiot, Richer, Veillette, Pausova & Paus, 2009; Blakemore, Burnett & Dahl, 2010). In animal models (e.g. Schulz, Richardson, Zehr, Osetek, Menard & Sisk, 2004), gonadal hormones have organizational and activational effects on limbic regions and association cortex. However, some adolescent neuromaturational effects are independent of puberty hormones. For example, adolescent patterns of changing dopamine receptor expression in the striatum of rats are preserved even in the absence of gonadal hormones (Andersen, Thompson, Krenzel & Teicher, 2002).

In humans, age and pubertal status are partly dissociable, with a 4–5-year normal variation in the timing of onset of puberty (Parent, Teilmann, Juul, Skakkebaek, Toppari & Bourguignon, 2003; Tanner & Whitehouse, 1976). Only a few functional neuroimaging studies of the adolescent brain have included puberty measures. One fMRI study demonstrated differences in caudate and rostral medial prefrontal BOLD signal between early and late puberty groups (aged 11–13) when processing reward outcome in a gambling task, and a correlation between testosterone level and caudate BOLD signal (Forbes, Ryan, Phillips, Manuck, Worthman, Moyles, Tarr, Sciarrillo & Dahl, 2010). A second fMRI study investigating reward and pubertal hormonal concentration showed a different, significant correlation between testosterone level and striatum activation (Op de Macks, Gunther Moor, Overgaauw, Güroğlu, Dahl & Crone, 2011). Two fMRI studies have been published assessing changes in face processing with puberty. One showed evidence for increased BOLD signal in the amygdala and ventrolateral prefrontal cortex to threatening faces in a pre/early puberty group compared with a mid/late puberty group (aged 11–13; Forbes, Phillips, Silk, Ryan & Dahl, 2011). In a different study, at 10 and 13 years, Moore and colleagues found that participants in later stages of pubertal development showed increased signal in face processing regions when looking at affective facial expressions (Moore, Phillips, Silk, Ryan & Dahl, 2012). These studies report some discrepant findings, which may reflect the different methods of assessing pubertal development used, or the different tasks administered (Op de Macks *et al*., 2011; Moore *et al*., 2012). However, no previous study has investigated pubertal influences on the ‘mentalizing network’ of the social brain.

One of the hallmarks of adolescent development is the dramatic change that occurs in social behaviours. Adolescents show heightened self-consciousness, develop increasingly complex and important peer relationships, experience sexual feelings and form romantic relationships, and demonstrate better understanding of other people compared to younger children (Steinberg & Morris, 2000; Spear, 2009). The emergence of these behaviours coincides with the physical changes of puberty, which prompts the hypothesis the social behavioural changes of adolescence result from increasing pubertal hormone levels, perhaps via a direct influence on brain structure and function (Forbes & Dahl, 2010). Mentalizing, the ability to recognize and interpret the feelings, intentions, beliefs and desires of others (Frith & Frith, 2003), is important for all of these social behaviours. For example, to experience self-consciousness, an individual must be aware of the perspectives and opinions of other people.

The network of brain regions recruited during mentalizing tasks comprises the dorsomedial prefrontal cortex (DMPFC), posterior superior temporal sulcus (pSTS) at the temporo-parietal junction (TPJ) and the anterior temporal cortex (ATC). Developmental studies have shown a shift in relative activity within regions of the mentalizing network between adolescence and adulthood (Blakemore, 2008). Specifically, a number of studies have shown that signal in the DMPFC during mentalizing tasks decreases with age across adolescence, while signal in temporal regions increases during the same period (e.g. Blakemore, den Ouden, Choudhury & Frith, 2007; Burnett, Bird, Moll, Frith & Blakemore, 2009; Pfeifer, Masten, Borofsky, Dapretto, Fuligni & Lieberman, 2009; Wang, Lee, Sigman & Dapretto, 2006). In the current study, our aim was to investigate the differential effects of chronological age and puberty status on brain activity during a mentalizing task, and specifically a task exploring the emotional sensitivity to opinons and actions that characterize early adolescence (Sebastian, Viding, Williams & Blakemore, 2010). We therefore used a ‘social emotion’ mentalizing task that we previously designed to investigate social brain development across age in females (Burnett *et al*., 2009), to investigate the impact of puberty on social emotion processing. Social emotions (e.g. guilt, embarrassment) are emotions that require mentalizing about others and their reactions to one’s actions; in contrast, basic emotions (e.g. disgust, fear) do not require mentalizing. Adolescent females aged 11–13 years performed the task during fMRI. This age range incorporates females at all pubertal stages and is characterized by steep gradients of gonadal hormone secretion (Rubin, Maisonet, Kieszak, Monteilh, Holmes, Flanders, Heron, Golding, McGeehin & Marcus, 2009).

Three independent measures of puberty were obtained: salivary hormone assays for testosterone, oestradiol and dehydroepiandrosterone (DHEA); visual clinician assessment of Tanner stage (Marshall & Tanner, 1969); and a self-report measure of menarcheal status. Tanner stage and menarcheal status were combined to define pre/early puberty and mid/late puberty groups, which were used for our analysis. We predicted that puberty measures would be related to Social>Basic activity within the ATC since this mentalizing region is densely connected with steroid hormone receptor-rich limbic regions (Ahmed, Zehr, Schulz, Lorenz, DonCarlos & Sisk, 2008; Cooke & Woolley, 2005). In contrast the DMPFC is thought to be sensitive to age effects, and not puberty (Casey, Duhoux & Malter Cohen, 2010). We thus hypothesized that chronological age would predict functional changes in DMPFC, independent of the effects of puberty.

## Methods

### Participants

The sample consisted of 42 female adolescents aged 11.1 to 13.7 years (mean 12.5, *SD* 0.7 years; see Table 1). Within this narrow age band, normally developing adolescent girls can be at any puberty stage from Tanner stage 1 to 5 (Marshall & Tanner, 1969), thereby providing maximal pubertal variability while minimizing variance in age. Participants were recruited via advertisements posted around the university campus and letters sent to local schools. Potential participants were excluded based on parent report if they had a history of previous neurosurgery, premature birth (<34 weeks gestation), a diagnosis of epilepsy, autistic spectrum disorder, dyslexia, known psychiatric disorder, or a known endocrine disorder. All participants had normal or corrected to normal vision and spoke English as their native language. Participants each assented to the study, and informed written consent was obtained from a parent/guardian. Subjects received £10/hour for their participation in data collection (max. 2 hours). The study was approved by the UCL National Hospital for Neurology and Neurosurgery Ethics Committee.

**Table 1:** Demographics showing mean, standard deviation and range of participants for age, BMI, vIQ, pubertal hormone levels and Tanner stage for the whole group (N = 42) and for the Early and Late puberty groups separately. Significant differences (p <.05) between puberty groups in bold. Age and vIQ were covaried out of subsequent analyses

	Whole group (*n* = 42)	Puberty groups		
		Early (*n* = 21)	Late (*n* = 21)	
	Mean ± *SD* (Ramge)	Mean ± *SD* (Range)	Mean ± *SD* (Range)	Difference between group means
Age	12.5 ± 0.7 (11.1−13.7)	**12.2 ± 0.7 (11.1−13.6)**	**12.9 ± 0.6 (12.0−13.7)**	**t_40_ = 3.47 *p* <.001**
BMI^ (*N* = 41)	19.2 ± 3.0 (13.5−27.3)	19.2 ± 3.5 (14.1−27.3)	19.1 ± 2.5 (13.5−24.6)	t_39_ = 0.039 *p* =.969
vIQ	120.9 ± 12.6 (89−155)	**116.6 ± 13.2 (89−139)**	**125.2 ± 10.6 (107−155)**	**t_40_ = 2.34 *p* =.024**
Oestradiol+ (*N* = 39)	3.60 ± 1.74 (1.34−9.86)	3.11 ± 0.90 (1.39−4.98)	4.06 ± 2.20 (1.34−9.86)	t_39_ = 1.91 *p* =.067
Testosterone+ (*N* = 41)	60.8 ± 23.7 (28.1−148.3)	53.9 ± 12.1 (30.9−78.3)	67.4 ± 29.8 (28.1−148.3)	t_37_ = 1.78 *p* =.088
DHEA+ (*N* = 40)	176.0 ± 107.1 (56.5−534.3)	150.5 ± 61.0 (56.5−304.3)	201.5 ± 135.8 (69.4−534.3)	t_38_ = 1.53 *p* <.134
Tanner stage breast	3.3 ± 1.2 (1−5)	**2.3 ± 0.6 (1−3)**	**4.3 ± 0.7 (4−5)**	**t_40_ = 10.19 *p* <.001**
Tanner stage pubic hair	3.1 ± 1.2 (1−5)	**2.1 ± 0.8 (1−3)**	**4.1 ± 0.7 (3−5)**	**t_40_ = 8.91 *p* <.001**

^ One girl in the Early puberty group was not measured for height, leaving N = 41 for BMI for the whole group, and N = 20 for the Early puberty group.

+ One subject did not produce a saliva sample. There was insufficient sample collected for one subject for analysis of either DHEA or oestradiol, and insufficient in a second participant for oestradiol only.

Verbal IQ (vIQ) was measured using the British Picture Vocabulary Scale II (Dunn, Dunn, Whetton & Burley, 1997), which was administered individually to participants in a quiet testing room. Body Mass Index (BMI) was calculated for each participant except one whose height was not measured (see Table 1).

### Endocrine assessments

Three independent measures of pubertal development were taken from each participant:

1. Salivary hormone assays for testosterone, oestradiol and DHEA. These are the principal hormones that drive the physical and behavioural changes of puberty. We used salivary hormonal assays rather than serological assays to minimize invasive testing. Upon waking on the morning of their scan, before 9am, each participant collected 2 ml passive drool (unstimulated) samples of saliva after rinsing their mouths with water, and before brushing their teeth, eating or drinking anything (except water). We verified that these instructions had been followed by parental report. The samples were transported on the day of collection to the testing centre on ice in an insulated box. Samples were stored at −80°C and later analysed simultaneously by Salimetrics Europe Ltd (http://www.salimetrics.com/).
2. A visual assessment of breast and pubic hair stage using established Tanner stages (Marshall & Tanner, 1969) by a trained paediatric physician (ALG). If a participant chose not to be examined (*N* = 2), they were asked to rate their own developmental stage using Tanner stage diagrams (Taylor, Whincup, Hindmarsh, Lampe, Odoki & Cook, 2001).
3. Self-report of menarcheal status and timing.

On the basis of (2) and (3), participants were dichotomized into pre/early puberty (referred to as Early) and mid/late puberty (referred to as Late) puberty groups. Participants were characterized as Early puberty if both breast and pubic hair Tanner stages were 1, 2 or 3 and if they were pre-menarcheal. Participants were characterized as Late puberty if either breast or pubic hair stage was 4 or 5 or they were post-menarcheal (Dorn, 2006). Early and Late puberty groups differed significantly on both age and vIQ (see Table 1); therefore, we included age and vIQ as covariates in all subsequent between group/hormone analyses.

### fMRI task

During the fMRI experiment, participants read scenarios designed to evoke one of four emotions: two social emotions (embarrassment and guilt) and two basic emotions (disgust and fear; see Burnett *et al*., 2009). Scenarios featured the protagonist (‘you’) plus one other person. Consequently, the crucial difference between social and basic emotion conditions was the requirement for mentalizing, not the mere presence of another person in the scenario (Abraham, Werning, Rakoczy, von Cramon & Schubotz, 2008). The mean (and range) word length, and the number of clauses, was equated across emotion conditions.

After reading each scenario, participants rated to what extent they would feel the given emotion, on a discrete rating scale from 1 (not at all) to 4 (very much), using a button box. Participants had 9 s to read silently, imagine and rate their response to each emotion sentence. There were 72 emotion scenarios in total, presented in blocks of three. In each block, all three scenarios featured the same emotion (disgust, embarrassment, fear or guilt). At the start of each block, a 1 s cue screen informed participants which emotion would be featured. Prior to scanning, all participants completed a guided practice session consisting of one example scenario from each of the emotions. The example scenarios did not appear inside the scanner.

The fMRI experiment was split into two 7 min sessions, each containing 12 emotion blocks, each lasting 28 s. Condition order was fully randomized. In addition there were four 7 s visual fixation blocks per session, occurring at regular intervals through both sessions. Stimulus presentation was programmed in Cogent (http://www.vislab.ucl.ac.uk/Cogent/index.html) running in Matlab 7.3.0, which recorded participant responses.

### Data acquisition

A 1.5T Siemens Sonata head MRI scanner with 8-channel phased-array coil was used to acquire 3-D T1-weighted fast-field echo structural images and multi-slice T2*-weighted echo-planar volumes with blood oxygenation level dependent (BOLD) contrast. Each functional brain volume was composed of 45 3 mm axial slices with a 1.5 mm gap and in-plane resolution of 3*3 mm, with −30° slice tilt, zero z-shim and negative (down) PE direction to minimize signal dropout in the orbital/rostral prefrontal and anterior temporal cortices. Repetition time was 4.05 s (90 ms per slice*45 slices). A total of 218 volumes were acquired over the two sessions, or 104/114 scans per session.

Prior to functional scanning we acquired individual field maps (scanning time 2 mins) to correct for distortions in functional images (Weiskopf, Hutton, Josephs & Deichmann, 2006). After functional scanning we acquired a 10 min T1-weighted anatomical image for each participant. The total scanning duration was approx. 30 mins per participant.

### Hormonal data analysis

Duplicate assays for testosterone, oestradiol and DHEA were performed for each participant, with intra-assay variation of <7% for all results. Therefore, the mean values were used for all analyses. Regression analyses were performed to assess the relationship between hormone levels and age, BMI and vIQ.

### Behavioural data analysis

Emotion ratings were analysed using 2 × 2 mixed model repeated measures ANOVA with between-subjects factor Group (Early vs. Late puberty) and within-subjects factor Emotion (Social vs. Basic). A regression analysis was performed to assess the relationship between mean emotion ratings and hormone levels.

### Functional imaging data analysis

Imaging data were analysed using SPM5 (http://www.fil.ion.ucl.ac.uk/spm). The first six volumes from each run were discarded to allow for T1 equilibrium effects, leaving 206 image volumes per participant. Preprocessing included rigid-body transformation (realignment) and unwarping using individual field maps to correct for head movement. The images were then stereotactically normalized into the standard space defined by the Montreal Neurological Institute (MNI) template using the mean of the functional volumes, and smoothed with a Gaussian filter of 6 mm full-width at half maximum to increase signal-to-noise ratio and facilitate group analysis. Time series for each participant were high-pass filtered at 128 s to remove low-frequency drifts.

The analysis of the functional imaging data entailed the creation of statistical parametric maps representing a statistical assessment of hypothesized condition-specific effects (Friston, Jezzard & Turner, 1994), which were estimated with the General Linear Model (GLM). The effects of interest were the two scenario block types (Social and Basic emotion) and the visual fixation blocks. We modelled the six realignment parameters as effects of no interest to account for any group differences in head movement. Mean movement across the scans was 0.41 mm (*SD* 0.21) for translation, and 0.40 degrees (*SD* 0.23). First-level contrast images ([Social>Fixation]>[Basic>Fixation], referred to as (Social>Basic) were initially examined to look for main effects across the whole group, and then were entered into four second-level (random effects) multiple regression models examining: (a) the association between neural activity related to Social>Basic emotion processing and each puberty hormone (testosterone, oestradiol and DHEA), controlling for age and vIQ; and (b) the relationship between neural activity related to Social>Basic emotion processing and age (controlling for each puberty hormone and vIQ). At the second level we also modelled the interaction between condition (Social>Basic) and puberty group (Early vs. Late). A priori regions of interest were investigated based on peaks reported in Burnett *et al*. (2009), which employed the same paradigm in a different sample of adolescents and adults, and showed main effects of the Social>Basic condition in the MPFC ([−10 52 18]; [−4 52 −8]; [−18 42 16]; [−16 48 34]), precuneus [4 −56 28; −4 62 40], left pSTS/TPJ [−38 −66 42] and right pSTS/TPJ[44 −48 28]. In the second-level analysis, Burnett *et al*. (2009) showed age-related changes in social emotion processing in the left ATC [−40 −6 −26] and the left DMPFC [−16 42 20]. We conducted small volume corrections (SVCs) on spheres with radius 6 mm centred on these previously reported peak activations. We report activations within these regions that survive family-wise error (FWE) SVC (*p* <.05) and, for completeness, activations that survive either cluster level FWE corrected threshold of *p* <.05 or whole brain FWE height threshold at *p* <.05. Brain mapping figures were made using Caret (Van Essen, Dickson, Harwell, Hanlon, Anderson & Drury, 2001).

## Results

Table 1 shows characteristics of subjects with respect to age, BMI, vIQ and salivary hormone levels, for the whole sample and for Early and Late puberty groups.

### Pubertal data

Physician-assessed Tanner staging data were available for 40 participants, with self-reported Tanner stage data for the remaining two participants.

Salivary hormone data were available on 42 participants. Mean levels were similar to previously reported norms for adolescents (Granger, Schwartz, Booth, Curran & Zakaria, 1999; Matchock, Dorn & Susman, 2007; Shirtcliff, Dahl & Pollak, 2009). There were significant correlations between both oestradiol and testosterone and Tanner stage of breast development, and between testosterone and Tanner stage of pubic hair development (all *p*s <.05) (see Table 2 for all correlations). We found no association between hormone levels and either age, vIQ or BMI.

**Table 2:** Correlations between pubertal measures, participant demographics and behavioural ratings showing Pearson r coefficients

	Tanner stage pubic hair	Tanner stage breast	Oestradiol	Testosterone	DHEA	Age	BMI	vIQ
Tanner stage breast	0.90***							
Oestradiol	0.31	0.44**						
Testosterone	0.40**	0.36*	0.50***					
DHEA	0.31	0.31	0.61***	0.69***				
Age	0.68***	0.58***	0.24	0.18	0.20			
BMI	0.15	0.13	0.01	0.03	0.20	0.18		
vIQ	0.26	0.41**	0.01	0.00	0.02	0.12	0.25	
Mean Basic rating	−0.22	−0.28	−0.02	0.02	0.07	−0.19	−0.14	−0.26
Mean Social rating	−0.16	−0.26	−0.14	−0.06	−0.01	−0.17	−0.13	0.25

*** *p*<.005

** *p*<.01

* *p*<.05.

### Behavioural data

Emotion rating data from four participants were not recorded by the stimulus computer, leaving *N* = 38. There were no correlations between mean emotion ratings and hormone levels after controlling for age and vIQ (all *p*s >.5).

Table 3 shows emotion ratings by scenario and puberty group. After co-varying out age and vIQ, there was a main effect of group: the Early puberty group gave higher ratings than the Late puberty group. There was no significant effect of emotion and no interaction between puberty group and emotion (all *p*s >.1).

**Table 3:** Mean emotion ratings by participants in Early and Late Puberty groups. There was a main effect of group: the Early puberty group gave higher ratings than the Late puberty group, which remained significant after age and vIQ were partialled out (F(1, 34) = 4.87, p =.034). There was no interaction between puberty group and emotion (F(1, 36) = 0.055; p >.816)

Emotion	Puberty group	Emotion rating Mean (*SD*)
Basic	Early puberty (*N* = 18)	3.34 (0.23)
	Late puberty (*N* = 20)	3.07 (0.36)
Social	Early puberty (*N*=18)	3.22 (0.25)
	Late puberty (*N*=20)	2.97 (0.31)

### fMRI data

#### Main effect of Social>Basic emotion processing across participants (*N* = 42)

**Figure 1:**
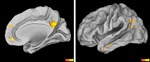
Main effect of Social>Basic emotion across the whole group (N = 42), showing activity in the DMPFC, precuneus (left image, medial view), bilateral pSTS/TPJ, bilateral ATC (right image, lateral view), shown at p <.001, minimum cluster size 10 voxels.

Across the whole group, the main effect of Social>Basic emotion was associated with BOLD signal change in the DMPFC, bilateral pSTS/TPJ, precuneus and bilateral ATC, as shown in Figure 1 (see Table 4).

**Table 4:** MNI co-ordinates, z-values and cluster size for main effect of Social>Basic emotion. We report activations that (a) survive FWE SVC (p <.05) within our a priori predicted regions (see Methods) or (b) show cluster level corrected threshold of p <.05. There were no clusters that survived whole brain FEW height threshold at p <.05

Whole group (*N*=42)	Region of activation	MNI co-ords	*z*	Size in voxels at *p*<.001
Social>Basic emotion	Precuneus	−2 −56 36	4.32	587^a,b^
	Superior DMPFC	22 38 50	4.36	133^b^
	VMPFC	6 50 −10	4.23	261^a,b^
	DMPFC	8 54 24	4.01	88^a^
		−6 48 32	3.51	23^a^
	pSTS/TPJ – left	−46 −60 38	4.09	214^a,b^
	pSTS/TPJ – right	56 −64 26	3.73	98^b^
	Lateral occipital cortex – left	−48 −82 6	3.81	94^b^

#### Relationship between hormones and social emotion processing

**Figure 2:**
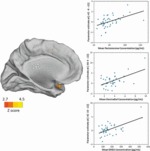
There was a positive association between level of puberty hormones and BOLD signal during Social>Basic emotion (with age and vIQ covaried out) in the left ATC (peak voxels: testosterone [−42 −8 −22]; oestradiol [−40 4 −22]; DHEA [−42 10 −22]). The overlapping region of activation is shown here at p <.005. The graphs on the right show the positive correlation at the peak voxel between puberty hormone and adjusted BOLD signal in the Social>Basic contrast in the left ATC for testosterone (r = 0.472); oestradiol (r = 0.532); DHEA (r = 0.607).

Regression of whole-brain BOLD response for the contrast Social>Basic emotion against testosterone revealed a cluster in the left ATC [−42 −6 −22]. This activation remained significant [peak voxel −42 −8 −22] after covarying out vIQ and age (see Figure 2 and Table 5). Oestradiol and DHEA concentrations were also positively correlated with activity within the left ATC, both in a stand-alone model and when covarying out vIQ and age (note that the correlations did not reach significance at SVC FWE correction; see Table 5). For all three hormones, incorporating the covariates vIQ and age did not change the level or significance of the clusters of interest, but did attenuate the size of the significant clusters (results shown including covariates).

**Table 5:** (a) Positive regression between hormones and BOLD signal during Social>Basic emotion processing in left anterior temporal cortex with age and vIQ partialled out (Note: analysis without covariates shows no qualitative change in results). (b) Negative regression between age and BOLD signal during Social>Basic emotion processing in medial prefrontal cortex with hormone levels (testosterone, oestradiol and DHEA) and vIQ partialled out

	Regressor	*N*	MNI co-ords	*z*	Size in voxels at *p* <.001
a.					
Positive regression	Testosterone*	41	−42 −8 −22	3.16	2
	Oestradiol#	39	−40 4 −22	3.38	6
	DHEA#	40	−42 10 −22	3.94	23
b.					
Negative regression	Age*	42	−16 50 22	3.83	34

* survives SVC at *p*<.05 FWE.

# survives SVC at *p*<.1 FWE.

#### Interaction between puberty group and social emotion processing

There were no regions that survived our significance threshold in these interaction contrasts. The analysis was also performed excluding the covariates (age, vIQ) from the model, and again there were no regions that survived our significance threshold. With a narrowed age range (11.5–13.5 years; *N*=30), there was no significant difference in age between Early and Late puberty groups. A repeated analysis with this subgroup again showed no regions that survived our significance threshold (data not shown).

#### Relationship between age and social emotion processing

**Figure 3:**
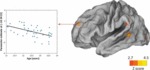
There was a negative association between age and BOLD signal during Social>Basic emotion (with puberty hormone level and vIQ covaried out) in the left DMPFC (peak voxel [−16 50 22]), shown here at p <.005. The graph on the left shows the negative correlation between age and adjusted BOLD signal at this peak voxel (r = −0.587).

Whole brain linear regression analysis between age and BOLD signal change during Social>Basic emotion revealed a negative correlation with age within the left DMPFC [−16 48 20]. This cluster remained significant after covarying out age and vIQ in the model (peak voxel [−16 50 22] with a small increase in cluster size (Figure 3; see Table 5).

## Discussion

In the current study, we used fMRI to investigate the relationship between puberty and the neural correlates of social emotion processing in females within a narrow age range from 11 to 13 years. We found evidence for functionally dissociable effects of puberty and age within the mentalizing network. Across the whole sample, we observed greater BOLD signal during social than during basic emotion processing in areas of the mentalizing network (DMPFC and pSTS/TPJ). This result is consistent with a previous study using the same paradigm in adolescent participants aged 11–18 and adults aged 22–32 years (Burnett *et al*., 2009). In our previous study, we observed an age-associated decrease in DMPFC activity during Social>Basic emotion processing, and an age-associated increase in the ATC. Our current results show puberty hormone-related, age-independent, increases in BOLD signal in the left ATC during social emotion processing. Conversely, we found chronological age-related decreases in BOLD signal within DMPFC that were not related to puberty hormone level.

### Puberty-related effects

We found significant associations between levels of testosterone, oestradiol and DHEA and BOLD signal in the left ATC during social relative to basic emotion processing. These relationships were independent of chronological age. These findings are consistent with the hypothesis that pubertal hormones interact with the neurocognitive changes seen during this time (Blakemore *et al*., 2010; Sisk & Zehr, 2005). There are a number of developmental mechanisms that could underlie the pattern of findings in ATC, and the current fMRI study cannot distinguish among these. First, the ATC is a paralimbic region with direct connections with limbic regions (Olson, Plotzker & Ezzyat, 2007), which contain large numbers of sex hormone receptors (Sar, Lubahn, French & Wilson, 1990; Tabori, Stewart, Znamensky, Romeo, Alves, McEwen & Milner, 2005). Thus, the increase in sex hormones at puberty might have a direct effect on activation of the ATC in social cognition tasks. Ernst and Mueller (2008), for example, demonstrated a relationship between adrenal hormone levels and amygdala activity during emotional face processing in females.

A second potential mechanism could be a developmental shift in cognitive strategy. The temporal poles, within the ATC, are thought to subserve semantic social knowledge (Lambon Ralph, Pobric & Jefferies, 2008; Olson *et al*., 2007; Zahn, Moll, Krueger, Huey, Garrido & Grafman, 2007). Transitions in social experience or cognitive strategy associated with increases in puberty hormone levels may cause a shift in the extent to which adolescents rely upon ATC representations during situations that provoke social emotions such as guilt and embarrassment. Previous studies have shown a relationship between puberty stage and emotion processing or more specifically, social emotion processing (Burnett, Thompson, Bird & Blakemore, 2011; Spear, 2009; Sumter, Bokhorst, Miers, Van Pelt & Westenberg, 2010). We previously investigated how the ability to understand social emotional scenarios using mixed emotions varied across puberty in girls aged 9–16 (Burnett *et al*., 2011). There was a change between early and late puberty in the number of emotional responses that participants gave in social emotion scenarios, with girls in late puberty attributing a wider combination of emotions in social scenarios than their peers in early puberty.

Note that, in the current study, we did not find a significant difference between the puberty groups (as classified by Tanner stage and menarche) on social brain activity, in the conventional analysis. This might be because Tanner staging is a noisier way of classifying individuals than measuring their puberty hormone levels. Nevertheless, the strong correlations between salivary pubertal hormone concentrations and physician-assessed Tanner staging suggest acceptable concurrent validity of our various methods. These intercorrelations are hypothesized to reflect the underlying physiological processes whereby puberty hormones drive physical development corresponding to distinct Tanner stages. Pubic hair development, driven by serum androgen levels in puberty, correlates well with our measure of salivary testosterone, whilst breast development, primarily controlled by oestrogens during puberty, has the highest correlation with salivary oestradiol levels. These robust intercorrelations suggest that hormonal concentrations used as continuous regressors for BOLD signal are a valid index of pubertal development.

The region of left ATC that was positively related to puberty hormones in the current study is in close proximity to the region of ATC previously found to show an *age*-related increase in activity during social vs. basic emotion processing, in a group of female participants aged between 11 and 32 years (peak voxels in the current study: testosterone [−42 −8 −22]; oestradiol [−40 4 −22] and [−36 −10 −22]; DHEA [−42 10 −22]; peak voxel in previous study [−40 −6 −26]; Burnett *et al*., 2009). In the current study, we found no age effects within this region across the narrow age range (11–13 years) of our sample. This raises the possibility that the interaction between age and emotion in ATC found in our previous study reflects predominantly pubertal changes, or a combination of age-dependent and pubertal changes.

Some behavioural patterns usually associated with adolescence have been shown to correlate more closely with pubertal maturation than age, including parent–child conflict (Steinberg, 1988), sensation-seeking (Martin, Kelly, Rayens, Brogli, Brenzel, Smith & Omar, 2002) and the development of romantic interests (Dahl and Spear, 2004). Our findings of puberty-related changes in neural activation, together with those shown in other recent fMRI studies using different ‘social’ tasks as described in the introduction, suggest that aspects of functional brain development in adolescence, like these behavioural changes, may be more closely linked to the physical and hormonal changes of puberty than chronological age.

Our study design focused on comparing brain regions activated during the social emotion condition versus the basic emotion condition, and did not include a baseline condition with which to compare these conditions. Thus the increase in ATC activation seen with advancing puberty associated with the Social>Basic contrast corresponds to an increasing difference in BOLD signal between social and basic emotion conditions as hormone level increases. The regression could be driven by increased activation during social emotion, decreased activation during basic emotion, or a combination of the two. Future studies might consider including an appropriate baseline condition (e.g. reading non-emotional control sentences) to allow a comparison between each of the two emotion conditions (social and basic emotions) and baseline to further explore this question.

### Age-related effects

We found an age-related decrease in activity within DMPFC during Social versus Basic emotion processing that was unrelated to puberty hormone concentration. This finding replicates the results from our previous study, which also showed an age-related decrease in DMPFC activity during Social compared with Basic emotion processing at very similar coordinates (peak voxel in the current study [−16 50 22]; peak voxel in previous study [−16 42 20]; Burnett *et al*., 2009). It is notable that an effect of chronological age was observed in the current study despite the very narrow age range (11–13 years) of the current sample; in the previous study, the age range was much wider (11–32 years). This adolescent decrease in DMPFC activity during social cognition tasks seems to be a robust finding in the literature: an age-associated decrease in activity within DMPFC during adolescence has been reported in nine developmental fMRI studies that have used a variety of mentalizing tasks (for meta-analyses see Blakemore, 2008, 2010). Recently, Gunther Moor *et al*. showed evidence that age-related differences in DMPFC activity during mentalizing were maximal in the transition from early adolescence (10–12 years) to mid-adolescence (14–16 years) in males and females, stabilizing thereafter (Gunther Moor, Op de Macks, Güroglu, Rombouts, Van der Molen & Crone, 2011). Therefore, it is possible that the early adolescent age range of our sample maximized our power to detect differences within a narrow age range.

There are a number of developmental mechanisms that could underlie this pattern of findings, and the evidence presented here cannot distinguish among these. An age-related shift in the cognitive strategy for social emotion processing might underlie the differences seen in DMPFC, which is thought to represent the mental states of self and other (Amodio & Frith, 2006). A number of studies have shown development during adolescence in behaviour during on-line or strategic social cognition tasks where participants have to take into account another’s mental state, either automatically or strategically (Dumontheil, Apperly & Blakemore, 2010; Güroglu, van den Bos & Crone, 2009). Alternatively, or in addition, BOLD signal change in this region could be due to age-dependent neuroanatomical maturation or neurovascular change (Andersen *et al*., 2002; Attwell, Buchan, Charpak, Lauritzen, Macvicar & Newman, 2010). Recent theoretical reviews have proposed that the cognitive operations subserved by dorsal prefrontal cortex mature with age, where age is shorthand for experience (Casey *et al*., 2010). According to this theory, more neurocognitive effort, requiring the recruitment of more extensive neural components, is needed to perform prefrontal-based cognitive operations at a younger age (Durston, Davidson, Tottenham, Galvan, Spicer, Fossella & Casey, 2006). The age-associated decrease within DMPFC that we observed was not related to puberty hormonal levels. Therefore, it is possible that decreases in DMPFC activity are driven by social experience, or the length of time an individual has been involved in social interactions. However, it should be noted that important life transitions such as puberty might cause abrupt changes in the rate of accumulation of social experience. Further work is needed to understand these complex relationships. The current set of findings suggests that changes during adolescence in social brain activity are not under the control of a single system. Instead, these changes may be differentially related to the effects of age and puberty, and may have multiply-specified biological and environmental drivers.

## Conclusion

We found evidence for a relationship in the ATC between puberty and the neural correlates of social emotion processing, independent of chronological age. Age, independent of puberty, was associated with activity in the DMPFC during social relative to basic emotion processing. The current study presents the first evidence of a functional dissociation between puberty status and age in early adolescence on activity within the mentalizing network.
